# Health and Non-Health Determinants of Consumer Behavior toward Private Label Products—A Systematic Literature Review

**DOI:** 10.3390/ijerph19031768

**Published:** 2022-02-04

**Authors:** Maksymilian Czeczotko, Hanna Górska-Warsewicz, Robert Zaremba

**Affiliations:** Department of Food Market and Consumer Research, Institute of Human Nutrition Sciences, Warsaw University of Life Sciences (WULS), 02-787 Warsaw, Poland; maksymilian_czeczotko@sggw.edu.pl (M.C.); robert_zaremba@sggw.edu.pl (R.Z.)

**Keywords:** consumer behavior, private label, health, perceived quality, systematic literature review, PRISMA

## Abstract

This study aimed to analyze the international literature on consumer behavior toward private label (PL) products, guided by the PRISMA (Preferred Reporting Items for Systematic Reviews and Meta-Analysis) method. We searched for peer-reviewed studies published until January 2021 in the Scopus and Web of Science databases using two main search terms, namely, “consumer behavior” and “private label,” which have several synonymous terms, such as “store brand,” “private brand,” and “own label.” A total of 44 eligible studies were selected for the analysis. We formulated research questions regarding the most studied categories of PL products, the non-health factors determining consumer behavior toward PL products, and the frequency of including health aspects in the choice of PL products. The following were analyzed in the studies included in the systematic literature review (SLR): general data and study design (authorship, year of publication, location, characteristics of the sample, and research category), research specifications (factors/variables, hypotheses, and measured parameters), and general findings (findings and practical recommendations). We found that most of the studies had analyzed dairy products as PL products, and the main non-health selection factors used were lower price and price–quality ratios. Health aspects were considered in only four of the analyzed studies, which focused on the evolution of PL products from low-cost products to sustainable brands with significant added value in terms of quality and health aspects.

## 1. Introduction

### 1.1. Health Aspects in Consumer Behavior 

Consumer behavior is increasingly being influenced by health aspects [1,2]. Consumers are becoming more aware of the need to eat healthy foods to maintain good health [2]. As a result, the quality of products is now considered to be as important as their price. Some consumers are willing to pay a higher price for products that guarantee high quality [3]. This can be linked with a greater understanding of health and the impact of food on health [4]. 

Currently, researchers show increasing interest in studying consumer decision-making styles in order to understand how people make purchasing decisions in a competitive environment [5,6,7,8,9,10,11,12,13,14,15]. Consumer behavior is influenced by several factors in the cultural, social, personal, and psychological realms, which together determine the basic attitudes and views of consumers, and which are also an important element of marketing [16]. According to Kotler and Keller [17], consumer buying behavior can be defined as the behavior related to how individuals, groups, and organizations acquire and dispose of goods, services, ideas, or experiences to meet their needs and desires. From the viewpoint of marketers, consumer behavior can be understood by analyzing the reasons why consumers buy, the factors influencing consumer buying patterns, the changing determinants within the society, and others [18]. The purchase of PL products is a personal choice, and the growing popularity of such products has gained the attention of retail researchers [19,20]. In particular, the development of premium and value PLs has affected consumption behavior, the final demands of consumers, and the shares held by other brands (national or local) [21,22].

Today, an increasing number of consumers are making informed purchasing decisions, including with regard to the brands offered by retailers. Consumers choose food by considering factors such as quality and nutritional value [23]. It has been proven that the health information provided on the label raises consumer awareness, and that health claims also influence consumer preferences and increase the likelihood of purchasing the product [24]. Because information is effective if it succeeds in meeting the specific needs of the target audience, understanding consumers’ information-seeking and -processing behavior is crucial for making better marketing decisions [25].

### 1.2. Evolution of PL Products and Consumer Perceptions

PL products are goods sold under the brand name of a retailer (i.e., supermarket, hypermarket, discount store) [26], or a name used exclusively as a brand of the retailer [27]. Several terms for PLs can be found in the literature and have been used in market reports on retailer brands. The main terms used for PLs are “private labels” [28], “private brands” [29], “private label brands” [30], “store brands” [31], “own brands” [32], and “own labels” [33].

Initially, consumers’ brand consciousness and preference for national brands (NBs) were perceived to be barriers to purchasing PL products, as they were considered to be of low quality [34]. Over the years, PL products have evolved as a result of product development in retail chains and changes in consumer preferences [35]. Four generations of PL products have been distinguished [36,37]. The first generation included undifferentiated core products, defined as generic, no-name, brand-free, or unbranded. They were sold under generic names and offered at a very low, competitive price. The second generation of PL products were defined as products of own brands or “quasi-brands,” and sold under the name of the retail chain. They stood out for their packaging and slightly higher quality, although it was comparatively lower than the market leader. The third-generation PLs, also known as own brands, were characterized by their names, which were analogous to existing manufacturer brand products. Their price and quality are comparable to those of leading producer brands. The fourth-generation PLs, called extended own brands, include innovative and differentiated products. Their price and quality were the same or higher than those of the products of leading manufacturer brands [36].

Distributors rank their PL products, most often, as economy, premium, or standard, based on their quality and price [38]. Standard PL products are generally considered to be medium-quality or medium-price alternatives of NB products [39]. In contrast, premium PL products are top-quality-tier products. Compared with NB products, these products are rated higher for their quality. Finally, economy PL products are of a basic acceptable quality at the best price and are lower in quality than the products of NBs [20].

It has been shown that consumers no longer perceive PLs as inferior in quality to NBs [40], and they are considered to have comparable quality [41]. In 2005, more than 70% of consumers in the US and Europe rated the quality of PL products as at least as good as the products of large brands [34]. In a survey conducted in 2015 in Poland, consumers indicated that the strength of PLs is their good quality–price ratio (64% of responses), next to lower price (83%) [42]. These findings were supported by our studies conducted in 2020 and 2021 in three European countries: Poland, the UK [43], and Spain (Tenerife) [44]. In our studies, respondents from countries with varying levels of development of PL products agreed that the quality of these products is high as well as comparable to manufacturer brands. Customers had a sense of trust and security when they shopped for PL products, and also valued these products for the wide collection and availability of retailers’ products. They also stated that PL products had the appropriate price–quality [43,44].

Studies indicate that the quality of PL products can be compared with the products of NBs, and thus these products can be treated as equal and highly competitive. However, the retailers must offer products with high quality at an attractive price in order to encourage consumers to buy [45]. Currently, most large retailers have labels that are becoming increasingly popular and trusted by customers [46]. Consequently, consumers show more positive attitudes toward PL products due to the increase in their quality as well as brand reputation, which is in line with the perception of consumers who feel good about purchasing PL products [47].

### 1.3. Aim of the Study

Our study aimed to analyze the international literature on consumer behavior toward PL products, guided by the PRISMA (Preferred Reporting Items for Systematic Reviews and Meta-Analysis) method. This study is the continuation of our previous research, which focused on the evolution of PL products into sustainable PL products in national markets with large PL market shares [43] and in an autonomous community, using Tenerife as an example [44].

We attempted to find answers to the following questions:

- What PL product categories have been studied in terms of consumer behavior?
- What are the non-health factors considered by consumers when choosing PL products?
- How often are health factors considered by consumers when purchasing PL products?

## 2. Materials and Methods

### 2.1. Study Design

We performed a literature search based on the PRISMA guidelines [48,49], which are widely applied in many academic studies [50,51,52,53]. Our search focused on studies published until 15 January 2021 in the Scopus and Web of Science databases.

### 2.2. Inclusion/Exclusion Criteria

Our systematic literature review (SLR) analyzed the international literature on consumer behavior toward PL products, including studies on the determinants of consumers’ choice of PL products, such as price, perceived nutritional values, economic factors, intentions, attitudes toward PL products, and packaging.

The studies that met the following criteria were included in the analysis: those based on empirical research and those describing consumer behavior toward PL products. Peer-reviewed papers were also included. No time limits were applied in the search of articles. We excluded publications written in a language other than English, papers presenting theoretical models, doctoral dissertations, editorials, book chapters, short reports, and conference publications, as well as articles for which full texts were not available.

### 2.3. Search Strategy

Studies were retrieved through a systematic search of peer-reviewed journals from two databases: Scopus and Web of Science. The search was conducted between 4 and 20 February 2021 and included articles that were published between 2000 and 15 January 2021.

To identify studies focusing on consumer behavior toward PL products, particularly food products, we used a combination of key terms in the search. The first term used was “private label products,” in various combinations and forms, and the second was “consumer behavior or preferences.” We used a search string in which separate groups of words were combined and then applied to both databases (Table 1).

A total of 150 and 100 studies were identified, respectively, in the Scopus and Web of Science databases. After eliminating duplicates, there were 197 studies. Following the review of titles and abstracts, 160 studies remained. The number of articles was then reduced to 99, and their eligibility was analyzed in depth by assessing the full text. Studies that were not written in English, those that did not focus on PLs, own brands, or store brands, or studies that did not relate to consumer behavior were excluded.

**Figure 1:**
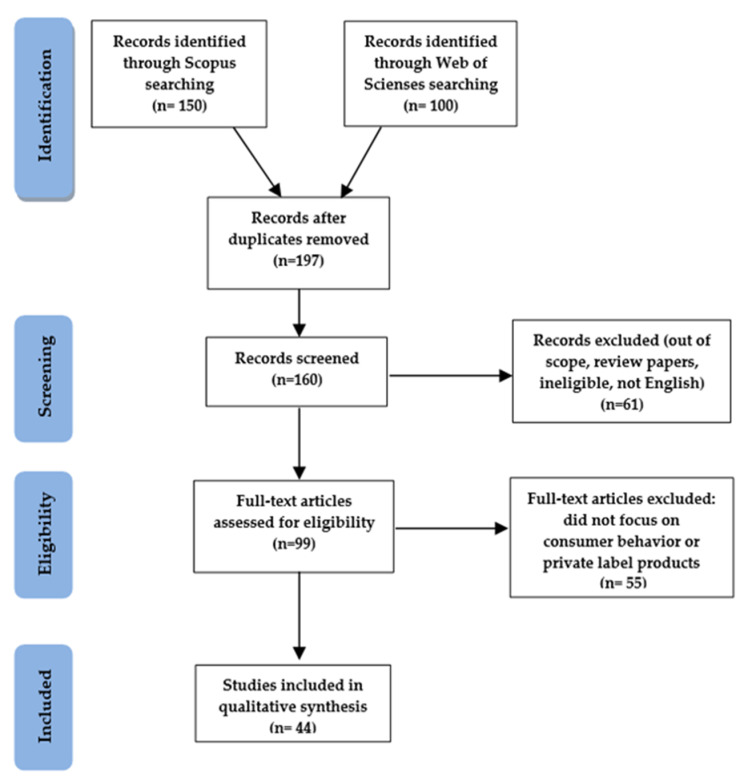
Identification, screening, eligibility assessment, and inclusion of articles in the systematic review (PRISMA). Source: [48,49].

Finally, 44 articles were selected for the analysis. Figure 1 presents a flow diagram describing the identification, screening, eligibility assessment, and inclusion of articles.

## 3. Results

All 44 studies included in the SLR were analyzed in three parts. The first part of the analysis focused on general information, including authorship, year of publication, research method used, country, sample population, product category, and the objective of the research (Table 2). The second part of the analysis focused on research specifications, which included the evaluation of factor/variables, hypotheses, and the types of data analysis used (Table 3). The third part of the analysis focused on key findings and practical implications of the studies (Table 4). In the Appendix A, in Table A1, we included the study objectives and research measures.

In all tables, studies are presented according to the year of their publication, starting with the most recent one (2021) and ending with the oldest (2000). To make the text analysis clearer in the tables, the retailer brand names are standardized by using the term “PL.” It also replaces other terms, such as store brand, private brand, private label brand, and own brand.

### 3.1. General Information

Table 2 presents general information pertaining to the studies included in the SLR.

The SLR included studies published between 2000 and 2021 as follows: seven studies from the period 2020–2021 [43,54,55,56,57,58,59], nine studies from the period 2018–2019 [16,60,61,62,63,64,65,66,67], 10 studies from the period 2015–2017 [38,68,69,70,71,72,73,74,75,76], six studies from the period 2010–2014 [29,77,78,79,80,81], and 12 studies from the period 2000–2009 [82,83,84,85,86,87,88,89,90,91,92,93]. The most frequently used research method was questionnaire survey (20 studies). The research sample consisted of between 200 [57,83] and 1272 respondents [61], but the average sample size was about 500. Other research methods used in the studies were experiments (six), in-depth interviews (six), blind sensory tests (four), scan panels (three), eye tracking (one), electroencephalography (two), and others (two). The studies included in the SLR had been conducted in cities located in Europe (31), America (eight), and Asia (six), as well as in Australia, New Zealand, and South Africa. The product categories mostly analyzed in terms of consumer behavior were dairy [29,43,54,60,63,79,80,81,82,88,93], cereals [16,43,54,55,56,58,68,69,73,76,80,85,88,92], sweets [16,43,54,60,63,79,80,81,82,85,88,90,91], frozen food [16,43,79,80,81,88], processed food [54,59,72,80,90], and cosmetics [16,38,60,70,74,75,76,79,81,87,90,91]. For example, in 2020, Slovak researchers conducted a series of studies on yogurts, which included a sensory comparison between PL products and products of NBs that are leading in the Slovak market [55,56,58]. Studies on nonfood product categories mainly chose cosmetics, especially shampoo, for the analysis of consumer behavior toward PL products [70,78,81,87,91].

### 3.2. Research Specifications

Table 3 presents the research specifications of the studies included in the SLR.

### 3.3. General Findings and Practical Implications

Table 4 presents the findings and conclusions from studies related to consumer behavior toward PL products, as well as managerial implications. The findings/conclusions mainly relate to how the studied factors, such as perceptions of quality, price, type of packaging, and risk of purchasing PL products, influence consumer behavior toward the PL products of retail chains. Practical recommendations are included in almost all the analyzed studies. Only one study did not provide any recommendations.

The main factors analyzed in the included studies were consumers’ perception of quality, price, store image, and the risk of PL products, and their attitude toward PL products in different forms. The other factors assessed were the risk of buying PL products in comparison to the products of NBs, the influence of the country of origin or packaging, and the effect of brand image and store chain on product choices. The results of the included studies were also supported by our studies conducted in Poland, the UK, and Spain (Canary Islands) on consumer behavior and the perception of PL products of retail chains in these countries. In all the three studies (the first two were carried out among Polish and British consumers [43], and the third one in Tenerife [44]), dairy products were rated highest in terms of the frequency of purchase of a given category of PL products.

Only four of the analyzed studies included health factors as determinants in the choice of purchasing PL products. The first study was performed in 2021, and proposes a new food labeling system with letter grades indicating the level of healthiness and recommended frequency of consumption of a product. Products were identified as healthier based on their Nutri-Score, and the healthiness of products, ranked across five categories, was evaluated differently. In addition, the study analyzed the impact of the Nutri-Score system on the perceived quality, perceived healthiness, and purchase intentions for NB and PL products. It also recommends that the Nutri-Score system can be introduced as the European nutrition label, and that it can be an effective option to manage the growing obesity epidemic [54].

In a second study from 2013, conducted in Germany, the researchers analyzed, through in-depth interviews, the four main motives for buying organic food: healthiness, hedonism, environmental friendliness, and food safety. The authors assumed that consumers have a belief that organic food has a higher nutritional value than nonorganic food, and has a higher degree of perceived healthiness compared to food from a brand without an organic label. The results confirmed that consumers perceived certified organic food to be significantly more healthy, hedonic, environmentally friendly, and safe compared to conventional or nonorganic food. This was also true in the case of organic PL products, which were ranked similarly to global organic brands by consumers. This indicates that consumers have positive perception toward organic PL products in terms of health aspects [29].

The third study analyzed the perceptions of manufacturer brands and PLs based on various choice factors. One of these factors was health, and respondents responded that PL products were comparable to the products of manufacturer brands, with a slight edge for manufacturer products, but this shows that consumers rate each brand equally, regardless of who owns it. This result could motivate retailers to further develop PL products, also taking into account the health aspects [84].

In the last study analyzed, which was conducted in 2006, the authors focused on the perceptions of purchase risk, comparing NBs and PLs for two nonfood products: shampoo and kitchen paper. The health aspects were discussed in the context of psychological risk during shopping, which was assessed by evaluating the level of fear caused by potential health harms. The results obtained were very similar, and supported the findings of other discussed studies that investigate the influence of health aspects on human health. The data showed that the greater the familiarity of consumers with PLs, the smaller the difference between PLs and NBs in terms of perceived risk, regardless of product category [87].

The studies included in the SLR used a variety of research methods. Quantitative research mainly used a survey questionnaire. Some studies conducted blind tests, in which consumers performed a sensory analysis of specific yogurt brands [55,56,58,68]. Most of the analyzed articles included research hypotheses (33), and a few included research questions [60,81,90], whereas some were devoid of both these research tools [43,54,68,71,74,80,85,88]. Only those research hypotheses that exclusively concerned PL products were taken for consideration in the analysis.

## 4. Discussion

We performed an SLR analysis on 44 studies related to consumer behavior toward PL products. The studies evaluated various factors determining the purchase of PL products, including perceived quality [54,55,56,59,65,69,72,73,82,83,85,88,89,91,92,93], packaging [55,56,69,73,86,93], price [29,38,59,63,64,69,70,71,72,73,74,75,77,78,82,83,86,88,89,90,91,92], health aspects [29,54,84,87], and brand loyalty [58,59,76,82,89]. Some of them also analyzed the frequency of the purchase of PL products [43,56,58,64,83,91].

The first research question concerned the product categories that were analyzed in the included studies. We found that the most analyzed food categories were dairy products, cereals, sweets, and frozen and processed food. These results reflect the value shares of product categories sold under PLs. For example, in western European countries, frozen foods (43%), chilled and fresh products (39%), and soft drinks (18.3%) have the highest value shares. As chilled and fresh foods, dairy products are frequently purchased by European consumers, and their value share ranges from as high as 55.4% in the UK to 42.1% in Spain and from 40.1% in the Netherlands to 21.8% in Italy [94]. In comparison, in the US, bakery products (36.6%), dairy products (33.1%), and delicatessen products (23.6%) had the highest share of sales in 2019 [95]. In Slovakia, dairy (40%) and durables (35%) were the most frequently purchased food categories, but the dairy category (46.6%) was dominant among products with the lowest income [16], and the sale shares of other categories were higher. Dairy products of PLs are therefore valued by consumers and selected by researchers for studies.

The available studies in the literature on PLs refer not only to consumer research. For example, studies conducted in Poland have analyzed PLs as a source of competitive advantage for international retail chains. It was found that organic PL products are competitive in terms of price, assortment range, variety, retailer image, sustainability and process uniqueness, and product-related attributes. The sales of organic PL products with offers allow consumers to buy organic food at more affordable prices and adopt a nutritious and sustainable diet with a low environmental impact [96].

The second research question concerned the non-health factors considered when consumers choose PL products. Our review shows that price is the main factor determining consumers’ choice of PL products. The significant influence of an attractive, lower price is confirmed by previous studies and reports on consumer behavior toward PL products [94,97,98,99]. The IRI report published in 2018 indicated that the average price of PL products in Europe in 2017 was about 70% of the average price of manufacturer brands, and these differences influenced consumers’ perception of PL products as low-cost products [100]. Such an image influences consumers’ price sensitivity, acting as a tool for building consumer loyalty to a retail chain and PL products [31,101]. This also highlights that PL products in general, as well as premium PL products specifically, are products of good value for money of [102]. Another frequently studied factor influencing the choice of PL products is the perceived quality of these products in comparison to NB products [103]. Many studies have analyzed the consumers’ perception of the quality of PL products. In reports and surveys, consumers have indicated a significant improvement in the quality of PL products. Importantly, the quality of PL products directly influences consumer loyalty to PLs and has an indirect impact on store loyalty [104]. Studies show that the quality of PL products is almost the same as that of NB products, which makes PL products more competitive. However, the retailers are required to maintain high quality at an attractive price in order to encourage consumers to purchase PL products [45]. This is also supported by the fact that consumers’ perception of higher quality increases their willingness to purchase PL products [105]. Our research in Poland and the UK showed that the high quality of products available under PLs is a more important factor for determining the purchase decision among UK consumers compared to Polish consumers [43]. At the same time, in the UK, the development of PLs is closer to sustainable and premium PLs, and quality improvement has become a key factor influencing choice [39]. Additionally, as indicated by a study in Germany, quality improvement has a stronger effect on the growth of PL market share compared to the case of NBs [106].

Although health aspects play an increasingly important role in consumer behavior toward PL products, they are not considered to be the main factor determining the choice of PL products. The inclusion of health considerations in consumer behavior toward PL products represents a gap in knowledge or research identified in this literature review. In answering the third research question, only four articles included in our SLR focused on health aspects. At the same time, the literature indicates the growing consumer awareness of food and its impact on well-being and health [1,2,4]. For example, the available research refers to different product categories, such as bread, fruit snacks [107], ready-to-eat cereals, and organic and functional foods [25], as well as food in general [108]. Research focusing on the consumer side addresses issues such as their willingness to eat bread with health benefits [109], the use of nutrition and health information on labels to increase the demand for bakery products [110], and the pleasure of eating and healthy food behaviors [111]. One study analyzed the attitudes of consumers toward healthy foods, with particular reference to organic and functional products that may contribute to better strategic and tactical marketing decisions, and which may also be used by government agencies in designing public health programs [25]. In one study conducted in the UK, US, and Germany, the impact of product attributes regarding the nutritional and health values of products on consumer choices was analyzed. European consumers were found to be more health-conscious in terms of lifestyle and diet than American consumers, and more focused on the nutritional value of the product, nutrition claims, or food labeling systems, rather than just the price and visual issues of product packaging [112]. Another study explored the perceptions of health by identifying elderly adults’ beliefs about food and health-related aspects, and showed that, according to senior consumers, health is about personal well-being (life is enjoyable) or about preventing diseases (energy and autonomy) [108]. In some studies, the authors examined consumer behavior in terms of health aspects, and found that consumers analyzed marketing activities, in particular marketing communication. For example, one of the studies analyzed the impact of two types of advertising content—healthy eating and anti-obesity—on the demand for healthy and unhealthy food products and beverages. The results indicated that among overweight consumers, anti-obesity advertisements were more effective than advertisements promoting healthy eating in reducing the demand for unhealthy items and increasing the demand for healthy products [2]. Some studies analyzed healthcare consumer behavior in online communities [113], the effect of product health information on consumer liking and choice [24], and the impact of health-promoting campaigns on sales [114].

Research related to the importance of health factors from the producers’ side indicates that there is a need to produce innovative products. These include healthy snacks for immediate consumption which are unique in terms of nutritional value and lack additives [107]. The need for innovative products is also indicated in studies on organic and functional foods [25], cereal products [115], and probiotic foods [116].

Our literature review fills the gap in the literature on the importance of health factors in consumer choices using the example of PL products. It has not only revealed the individual factors that have been analyzed by studies over time for selected product categories, but also shows the significance of health factors in private labeling and the different ways in which studies have analyzed consumer behavior toward PL products. The attention paid to the health aspects of PL products points to the development of PLs, characterized by a similar level of quality and price compared to producer brands. This increases the competitive rivalry in the market, and at the same time, for retail chains, provides a competitive advantage in strengthening their position in the market. In this way, PLs have reached the fourth generation of their development, which implies that analogous methods of brand creation, brand positioning and, above all, brand quality are evaluated by consumers at the same, or an even higher level.

Our study has some limitations. One of them is related to the fact that we excluded theoretical publications, conference materials, books, dissertations, and the reports of market research agencies, and included only publications in English in the SLR. Further research is needed as PL products continue to evolve into sustainable products. It is important to understand the intentions of retail chains regarding the development of PL products in order to verify if they are in line with the growing consumer awareness of the health aspects of food and nutrition. This will help in developing products under retail chains’ PLs with a high nutritional value based on nutritional recommendations.

## 5. Conclusions

Our literature review revealed that many factors influence consumer behavior toward PL products. The main non-health factors are price, quality, packaging, and purchase frequency of PL products, and brand loyalty. The perception of health factors was not among the frequently analyzed selection criteria, which may be due to the evolution of PL products from low-cost products to the products of sustainable brands. This review showed the changing issues related to researchers’ perceptions of the PLs of retail chains. Studies conducted at the beginning of the 21st century mainly analyzed price and its influence on PL product purchases. This was followed by value for money, and research in recent years has been focusing on premium and value-added products among PLs. Consumers have started to perceive these products as high-quality, innovative products, with organic packaging and health benefits. For the further development of PLs, an appropriate approach by retail chain managers is essential. Our review has identified several practical recommendations for designing new products, improving the quality of existing products in terms of raw material quality, packaging, design, and labeling, as well as developing effective marketing strategies, and monitoring consumer behavior and preferences. At the same time, expanding the PL product range with health-oriented, organic, innovative, and targeted products increases the competitive advantage of retail chains. This may allow for the availability of PL products as products sold for health reasons, which will align with the recommendations for healthy eating, proper diet composition, and choosing the right food.

## Figures and Tables

**Table 1:** Databases and terms used in the study and the number of results obtained.

Database	Search String
Scopus	TITLE-ABS-KEY (“private labels” OR “private label” OR “private label brands” OR “private brand” OR “own label brand” OR “own brand” OR “store brand”) AND TITLE-ABS-KEY (“consumer behaviour” OR “consumer behavior” OR “consumer preferences”)
Web of Science	TOPIC (“private labels” OR “private label” OR “private label brands” OR “private brand” OR “own label brand” OR “own brand” OR “store brand”) AND TOPIC (“consumer behaviour” OR “consumer behavior” OR “consumer preferences”)

**Table 2:** General details and design of the studies included in the systematic survey.

Author, Year	Research Method	Country	Sample Population	Product Category
Temmerman et al. (2021) [54]	Online experiment, survey	Belgium	796 respondents (students and employers of university) Study 1: pretest *n* = 52 and main study: *n* = 303 Study 2: *n* = 441	Study 1: 3 ready-to-eat meals Study 2: 20 products, including beverages, cookies, dairy products, meat and cereal products, fish, preserves
Kadekova et al. (2020) [55]	Study 1: survey with questionnaires Study 2: blind test 2 traditional + 3 PL yogurts	Slovakia	Adults ≤25 years Study 1: *n* = 549 respondents Study 2: *n* = 20 respondents	Dairy products: yogurts
Czeczotko et al. (2020) [43]	Survey with questionnaires distributed in a consumer panel, computer-assisted web interview (CAWI) method	Poland, UK	Adults ≥18 years declared to purchase PL food products *n* = 1000: 500 in Poland and 500 in the UK	Food products: dairy, grain products, sweets, biscuits, bakery products, meat products, fruit and vegetable products, frozen food, beverages, water, alcohol
Anitha and Krishnan (2020) [57]	Questionnaire survey, quota sampling method	India	Adults ≥18 years *n* = 200 respondents	n.a.
Košičiarová et al. (2020) [58]	Questionnaire survey, CAWI method, blind test: 2 traditional yogurts and 2 PL yogurts	Slovakia	Adults ≥18 years Survey: *n* = 693 respondents Blind test: *n* = 100	Dairy products: yogurts
Singh and Singhal (2020) [59]	Survey	India	Adults ≥18 years from 325 households who visited Big Bazaar Store	Sauces, preserves, ketchup, atta, mustard oil
Košičiarová et al. (2020) [56]	Questionnaire survey, blind test	Slovakia	Survey: *n* = 1116, ≥18 years Blind test: *n* = 20, ≤25 years	Dairy products: yogurts
Prediger et al. (2019) [60]	Half-factorial laboratory experiment, online survey	Spain	Adults ≥18 years *n* = 406 respondents	Fruit, vegetables, meat, fish, olives, cereals, bread, chips, sausages, beverages, gels, perfumes, detergents
Gómez-Suárez et al. (2019) [61]	Online survey based on Schwartz’s value conceptual framework model	USA, France, Germany, UK, Italy, Spain	Adults ≥18 years, *n* = 1272 shoppers buying FMCGs	n.a.
Salazar-Ordóñez et al. (2018) [62]	Online survey (household panel)	Spain	Buyers aged ≥19 years *n* = 1029 consumers	Olive oil
Liu et al. (2018) [63]	Study 1: simulated shopping, Study 2: questionnaires, Positive and Negative Affect Schedule scale, Study 3: behavioral lab	USA	Students: 570 respondents Study 1: *n* = 88; Study 2: *n* = 228; Study 3: *n* = 254	Fruit juice, canned vegetables, peanut butter, canned fruit, pasta, salad dressing, cereal products
Valaskova et al. (2018) [16]	Online survey	Slovakia	Adults ≥18 years *n* = 347 respondents purchasing PL products in one of the retail chains’ markets	Dairy products, baby food, durable goods, beverages, frozen food, cosmetics, sweets, detergents, animal food
Vázquez- Casielles and Cachero-Martinez (2018) [64]	Panel data with information about customers, data set: 187 weeks	Spain	Adults ≥18 years *n* = 254 regular customers	Fruit products: jam, 3 PL tiers (standard, economy, and premium) and NBs with share >5%
Garczarek-Bąk (2018) [65]	Eye tracking, electroencephalography, survey, CAWI method	Poland	*n* = 16 healthy right-handed respondents (8 female, 8 male) 21–30 years	10 (product categories) × 6 (brands) × 2 (variants): 7 categories of food and 3 categories of body care products and 6 products from different retailers
Meliana (2018) [66]	Questionnaire survey	Indonesia	260 shoppers in Indomaret and Alfamart	Groceries and household PL product category
Modica et al. (2018) [67]	Tactile exploration, visual exploration, visual and tactile exploration	Italy	Experiment 1: *n* = 19 Experiment 2: *n* = 13	2 daily food items (1 major brand and 1 PL) and 2 comfort food items (1 foreign product and 1 local product) 4 different comfort foods (e.g., chocolate bars) and 4 different daily foods (e.g., rice): 2 local and 2 foreign products of NBs and PLs
Schouteten et al. (2017) [68]	Sensory analysis, 3 sessions, online questionnaires	Belgium	Adults ≥18 years, *n* = 99 volunteers for sensory and consumer research (45 males and 54 females)	5 strawberry-flavored yogurts
Jara et al. (2017) [69]	Questionnaire survey	France	Adults ≥18 years Total *n* = 568 respondents: group A: *n* = 142, group B: *n* = 179, group C: *n* = 95, group D: *n*=152	Plain yogurts or a face cream
Gomez-Suarez et al. (2016) [70]	Online survey	Spain, Germany, France, UK, Italy, USA	Adults ≥18 years 1118 consumers of FMCGs from 6 countries (each *n* = 200)	Cosmetics: shampoo
Marques dos Santos et al. (2016) [71]	Save Holdings or Purchase task with functional magnetic resonance imaging, 64 blocks	Portugal	Adults ≥18 years *n* = 22 respondents buying NB and PL products (6 males and 16 females)	n.a.
Thanasuta (2015) [72]	Questionnaire survey	Thailand	Adults ≥18 years *n* = 240 shoppers of 5 hypermarkets and supermarkets in Bangkok	Cooking oil, tissue paper, body lotion, instant noodles
Schnittka (2015) [38]	Questionnaire survey	Germany	Adults ≥18 years *n* = 238 German consumers who were aware about PL products	Mineral water, detergents, juice, shower gel
Monnot et al. (2015) [73]	Experiment: 2 (overpackaging: present vs. absent) × 2 (brand concept: generic vs. mimic PL), face-to-face survey	France	Adults ≥18 years *n* = 217 consumers	Dairy products: yogurts
Diallo et al. (2015) [74]	Questionnaires from two retail chains during the shopping	Brazil	Adults ≥18 years *n* = 600 shoppers from 2 retail chains (Carrefour, Extra)	Cosmetics: shampoo
Zielke and Komor (2015) [75]	Online questionnaire	Germany, Poland	Adults ≥18 years *n* = 500 students (250 from Germany and 250 from Poland)	Groceries, consumer electronics, cosmetics, clothes
Fall-Diallo et al. (2015) [76]	Marketing scan behavior panels, purchase records, lasting 286 weeks: initial period (weeks 1–130), expansion period (weeks 131–208), and crisis period (weeks 209–286)	France	Carrefour customers who made at least two purchases in the analyzed period, butter data of 94 households: 869 purchases (expansion) and 888 purchases (crisis) yogurt data of 169 households: 2604 purchases (expansion) and 3368 purchases (crisis)	Dairy products: butter and yogurt
Delgado-Ballester et al. (2014) [77]	Mall intercept questionnaire survey	Colombia	Adults ≥18 years *n* = 600 shoppers who bought PL products during last 2 months (Carrefour and Éxito supermarkets)	Sugar, shampoo, facial cream, fabric conditioner, antibacterial gel, sunflower oil
Bauer et al. (2013) [29]	Study 1: in-depth interviews: main purchasing motives for organic food, Study 2: experiment: impact of organic label (OL) on consumer perception, Questionnaire: purchase intentions of buying organic PL products, Study 3: impact of OL on variables of behavioral intention analysis of OLs.	Germany	Adults ≥18 years Study 1: *n* = 12 German consumers using the laddering technique, Study 2 and 3: *n* = 630	Cereals
Fall Diallo et al. (2013) [78]	Self-administered questionnaires	France	Adults ≥20 years *n* = 266 respondents responsible for purchasing	n.a.
Herstein et al. (2012) [79]	Survey: questionnaire online	Greece, Israel, Portugal, Turkey	*n* = 683 undergraduate college students who purchase PL products	Chocolate, cooking oil, biscuits, rice, frozen meat, detergent, shampoo, toothpaste, liquid soap, and dishwasher liquid
Wyma et al. (2012) [80]	Survey: a structured questionnaire	South Africa	Adults ≥18 years *n* = 620, 4 supermarkets in an urban area	25 products, including dairy and cereal products, canned vegetables, frozen vegetables, beverages, sweets, oil, toiletries
Tifferet and Herstein (2010) [81]	Paper questionnaires	Israel	Adults ≥18 years *n* = 400 PL customers: students from 8 universities and colleges	Chocolate, laundry powder, oil, toothpaste, hummus, shampoo, frozen meat, liquid soap, rice, barrage bags
Glynn and Chen (2009) [82]	Mall intercept survey in city supermarket, screening question about purchase of 1 of 10 product categories with a PL offering	New Zealand	Adults ≥18 years *n* = 600 shoppers buying PL products	Canned fruit, toilet tissue, fresh milk, cheese, fruit juice, potato chips, biscuits, bread breakfast cereal, pet food
Anchor and Kourilová (2009) [83]	Structured questionnaires	Czech, Republic, UK	Adults ≥18 years *n* = 200 Tesco supermarket customers in the Czech Republic (*n* = 100) and the UK (*n* = 100)	n.a.
Kara et al. (2009) [84]	Self-administered questionnaires hand-delivered to respondents	USA	Adults ≥18 years *n* = 799 shoppers responsible for grocery shopping in the household	Grocery products
Albayrak and Aslan (2009) [85]	Face-to-face questionnaires on consumer preferences regarding private and manufacturer brand products	Turkey	Adults ≥18 years *n* = 217 consumers divided into 2 groups as those who buy PL products and those who buy NB products	Meat and dairy products, fruit and vegetables, sweets, oil products, wine
Cheng et al. (2007) [86]	Questionnaire survey	Taiwan	Adults ≥16 years *n* = 254 respondents	Various types of product categories
Mieres et al. (2006) [87]	Personal interviews	Spain	Adults ≥18 years *n* = 436 respondents buying kitchen rolls, *n* = 422 respondents buying shampoo	Kitchen rolls and shampoo
Akbay and Jones (2005) [88]	Supermarket scanner data, 65 weeks of observations	USA	100,000 consumers buying in 6 supermarkets: 3 stores chosen for primarily lower-income shoppers, and 3 stores that primarily serve consumers with higher income	Milk, breakfast cereals, ice cream, cooking oil, salty snacks, salad dressing, pasta, frozen vegetable, mayonnaise
Kurtulus et al. (2005) [89]	Face-to-face interviews with consumers who shop at the four major retailers	Turkey	Adults ≥20 years *n* = 514	n.a.
Semeijn et al. (2004) [90]	Experiment, online questionnaire consisting of 110 statements	The Netherlands	Students ≥18 years *n* = 128	Wine, toothpaste, potato chips, canned tomatoes
Veloutsou et al. (2004) [91]	Self-administered questionnaires, in-depth interviews with 5 consumers in each country to better interpret the results	Greece, UK (Scotland)	Adults ≥25 years *n* = 328 respondents: 104 from Greece and 224 from Scotland	Coffee, biscuits, toothpaste, liquid, shampoo
Miquel et al. (2002) [92]	Questionnaires in the form of personal interviews, each of the interviewed was valuing 2 of the 6 product categories	Spain	Adults ≥18 years *n* = 400 household shoppers	Milk, sliced white bread, oil, beer, bleach, toilet paper
Vaidyanathan and Aggarwal (2000) [93]	Experiment in 2 versions: visual stimulus with added branded or no-branded raisins; questionnaire booklet	USA	Adults ≥18 years Total sample: *n* = 175, *n* = 67 students and shoppers	Breakfast cereal with raisins

**Table 3:** Research specifications of the studies included in the systematic survey.

Author, Year	Factor/Variable	Hypotheses
Temmerman et al. (2021) [54]	Study 1: Perceived quality (PQ) Perceived tastiness (PT) Perceived healthiness (PH) Purchase intentions (PI) Study 2: Perceived healthiness (PH) Purchase intentions (PI) Nutritional knowledge (NK) Perceptions of healthy food (PhF) Dieting behavior (DB) Familiarity with Nutri-Score (NS) (FNS)	n.a.
Kadekova et al. (2020) [55]	Questionnaire: perception of PL product quality Blind test: sensory evaluation of yogurt, including color, aroma, consistency or density, taste and proportion of chocolate, the size of the packaging and its attractiveness	Gender (G) → buying PLs (–) G → quality rating of PLs (+) G → perception of PL product packaging (–) G → purchase of PLs (–) G → decisive factor to buying PLs (+) G → discouragement from buying PLs (–)
Czeczotko et al. (2020) [43]	Period of purchase of PL products (PP) Factors for purchasing PL products (FP) Opinions on the current development of PL products (OCD) Frequency of PL product purchasing (FPC) Share of PL products to total food purchases (SPL)	n.a.
Anitha and Krishnan [57]	Personal factor (PF) Impulse buying behavior (IBB) Store factor (SF) Urge to buy (UB)	PF → IBB (+); PF → UB (+) SF → IBB (–) SF → UB (+) UB → IBB (+)
Košičiarová et al. (2020) [58]	Purchase and frequency of purchase Brand loyalty Brand preference (traditional or PL) Motives for purchase Sensory properties of yogurts	Age → kind of preferred brand of purchased yogurts (+) Gender → kind of preferred brand of yogurts (+) A statistically significant difference in the purchasing preferences based on packaging (–) A statistically significant difference in the evaluation of yogurt flavors (+)
Singh and Kumar Singhal (2020) [59]	Perceived quality of PLs (PQ) Price consciousness (PC) Perceived value of PLs (PV) Store loyalty (SL) Quality consciousness (QC) Loyalty to PLs (PLL) Price sensitivity (PS) Willingness to pay for PLs (WP)	PQ → WP (+) PS → WP (–) PQ → PLL (+) PV → PLL (+) PV → the store’s overall image, in terms of brand and value (+) PLL → SL (+) PQ → SL (+)
Košičiarová et al. (2020) [56]	Questionnaire: Frequency of PL purchase (FPL) Purchases of PLs (P) Perception of quality (PQ) Consumer perception and consciousness about Product categories (CPC) Evaluation of packaging attractiveness (EPA) Factors of PL purchase (FP) Blind test: 7 chocolate-flavored yogurt samples; traditional brands vs. PL; investigated identical products	Gender (G) → PQ (+) G → P (+) Economic activity of respondents I → P (–) G → perception of PL product packaging (–) G → perception of facts that influence respondents to buy PLs (–) Age (A) → perception of facts that influence to buy PLs (–) G → decisive factor when buying PLs (+) R → decisive factor when buying PLs (–) G → facts that discourage from buying PLs (+) A → facts that discourage from buying PLs (+)
Prediger et al. (2019) [60]	Creating fictitious flyers and supermarket, featuring real NBs and fictitious PLs Different flyer designs (scenarios): (1) Store flyer page length; (2) Brand (NB or PL) on the cover page; and (3) An institutional slogan on the cover page as an incentive advertising Consumers received the flyers and answered an online survey Intentions to buy PL products	Four models: Scenario (S) I (NB on the cover, 8 pages, without a slogan) SII (PL on the cover, 20 pages, without a slogan) SIII (PL on the cover, 8 pages, with a slogan) SIV (NB on the cover, 20 pages, with a slogan)
Gómez-Suárez et al. (2019) [61]	Category: (1) Self-enhancement: self-transcendence, openness; conservation (2) Smart shopper self-concept (SSSC): smart-shopper behaviors, smart-shopper feelings, brand attitude (NB/PL)	Value structure (+) → attitude toward NBs (–) Value structure (+) → attitude toward PLs (–) SSSC (+) → attitude toward NBs (+) SSSC (+) → attitude toward PLs (+) Effect of SSSC on attitude → more positive for NBs than for PLs (+)
Salazar- Ordóñez et al. (2018) [62]	Attitude toward extra-virgin olive oil (EVOO) (AE) Attitude toward refined olive oil (AR) Perceived value of PLs (PV)	PV → AE (–) PV → AR (+)
Liu et al. (2018) [63]	Study 1: BESC (brand engagement in the self-concept); PL attitude; value consciousness; price consciousness Study 2: manipulated test in laboratory Study 3: manipulating brand engagement	Consumers with higher BESC prefer NBs over PLs (+) Consumers with lower BESC show increased preference for NBs relative to PLs (–) Consumers with higher BESC show reduced preference for NBs relative to PLs (+)
Valaskova et al. (2018) [16]	Consumer’s attitude (CA) and preferences in the choice of 10 categories of PL products	CA and individual demographic determinants (–) CA and factors leading to the purchase of PL products (–) CA and a particular type of the purchased product (–)
Vázquez- Casielles and Cachero- Martinez (2018) [64]	Information about products’ category (jam) and purchase situation: purchased brand, sale format of the purchased brand, purchased quantity, sale price, the product was on promotion, assortment size, and date of the last purchase	Economy PLs (EPL) → a negative brand-type similarity effect → decreases the choice of standard PLs (SPL) (–) EPLs → positive attraction effect → increases the choice probability of SPLs (+) EPLs → positive compromise effect → increases the choice probability of second-tier NB and SPLs (–) Premium PLs (PPL) → negative brand-type similarity effect → decreases the choice probability of EPLs and SPLs (+) PPLs → negative quality-tier similarity effect → decreases the choice probability of premium-quality NBs and second-tier NBs (+) PPLs → positive attraction effect → increases the choice probability of premium-quality NBs (+)
Garczarek-Bąk (2018) [65]	Perceived product esthetic (PPE) Perceived likelihood of buying the product (PI) Quality assessment (QA) Variants without showing the price and with normal price to control for the meaning of this factor	Women possess a relatively greater esthetic sensitivity to the appearance of PL products than men (–) The price knowledge will not affect the purchase decision of PL products within retailers (+) Young customers’ behavior in the process of buying PL products of distributive networks can be highly affected not by declared, but by latent factors (+)
Meliana (2018) [66]	Factors: logo, color, policy, cost, large stock, promo variations, complete products, and others	PL products have a significant effect on customers’ shopping preference PLs have a significant effect on store image
Modica et al. (2018) [67]	Comfort food vs. daily food Major brand vs. PLs Foreign vs. local Tactile, visual, and visual and tactile exploration	Major brand products present more attractive packaging than other products, and therefore elicit a higher approach tendency than the PL items (–)
Schouteten et al. (2017) [68]	Yogurt brands: two premium brands and three PLs Experiment: central location tests (*n* = 53) and home-use tests (*n* = 46) 3 test sessions (blind, expected, and informed)	-
Jara et al. (2017) [69]	Attitude (A) Perceived quality (PQ) Perceived price (PP) Packaging (P) Intent to buy (IB) Economic store brand (ESB) Organic store brand (OSB) Purchase intentions (PI)	PQ of PL products varies according to the type of P (+) Reinforced P → PQ of EPLs (+) Simplified P → PQ of EPLs (–) Simplified P → PQ of OPLs (+) Reinforced P → PQ of OPLs (+) Influence of PQ on the customers’ IB varies based on P (+) PQ of EPLs → PI due to a reinforced P (+) PQ of EPLs → PI due to a simplified P (–) PQ of OPLs → PI due to P (+) HPQ of OPLs → PI due to P (–) The more the type of P corresponds to a PL products’ positioning, the more it strengthens the customers’ IB (+) EPLs can increase customers’ IB via reinforced P (+) OPLs can increase customers’ IB via simplified packaging (+)
Gomez-Suarez et al. (2016) [70]	Two shampoo brands (NB and PL); different prices Preference (P) Attitude (A) Purchase intention (PI) Consumer preferences (CP) Quality inferences (QI) Smart shopper self-perception (SSSP) Consciousness (C)	A of PL products → preference for PL products (–) CP for PL products → PL products (–) C → A of PL products (+) SSSP → A of PLs (+) Familiarity with the NBs negatively(-) affects A of PLs (+) Perceived risk has a (−) impact on CP for PLs (+) C propensity for exploration has a (–) effect on PL product P (+) Impulsiveness has a (+) impact on PL product PI (+) QI made from price have a (–) impact on PL product A (+) QI made from brand image have a (–) impact on PL product A (–) QI made from brand reputation have a (–) impact on PL product A (–) QI made from product efficiency have (+) impact on PL product A (+)
Marques dos Santos et al. (2016) [71]	Analysis: product, price, decision, and interval 7 categories of food products (4 retailers × 7 categories = 28 different products × 2 brands (NB or PL)) Price manipulation applied	-
Thanasuta (2015) [72]	PL purchase Price consciousness (PC) Quality consciousness (QC) Brand consciousness (BC) Value consciousness (VC) Risk perception (RP)	PC → PL purchase (+) QC → PL purchase (−) BC → PL purchase (−) VC → PL purchase (+) RP → PL purchase (−) Product differentiation, risk level → PL purchase (+)
Schnittka (2015) [38]	1. Perceived brand (in low and high category) 2. Price preference 2 × 3 × 3: (a) Economy PLs (EPLs): low-priced store, high-priced store, and overall, for each category: manufacturer, retailer, overall (EPL) (b) Premium PLs (PPLs): low-priced store, high-priced store, and overall, for each category: manufacturer, retailer, overall (PPL) Consumer preferences (CP)	In low-priced grocery stores, EPLs evoke more favorable CP than PPLs (+) In high-priced stores, EPLs evoke less favorable CP than PPLs (+) In product categories of low brand relevance, EPLs evoke more favorable CP than PPLs (+) In product categories of high brand relevance, EPLs evoke less favorable CP than PPLs (+) If consumers believe that the PLs are produced by a well-known manufacturer, EPLs evoke more favorable CP than PPLs (–) If consumers believe that the PLs are produced by the corresponding retailer itself, EPL products evoke less favorable CP than PPLs (–)
Monnot et al. (2015) [73]	1. Price sensitivity (PS) Perceived quality (PQ) Environmental consciousness (EC) Perceived expensiveness (PE) Product involvement (PI) Perceived environmental friendliness (PEF) Perceived convenience (PC) 2. Mean with overpackaging (OP) and without overpackaging for mimic or generic PL products (yogurt)	Eliminating OP reduces PQ (–), reduces PE (+), increases PEF (+), and reduces the PC of the product (+) The influence of eliminating OP on the product’s PQ (+), PE (–), PEF (+), and PC depends on the PL concept: it should be stronger for a mimic PL product than for a generic PL product (+) The influence of eliminating OP on purchase intention is mediated by the product’s PQ (+), PE (–), PEF (–), and PC (+)
Diallo et al. (2015) [74]	Store image perceptions (SIP) PL price image (SPI) PL perceived value (PV) PL attitude (A) PL purchase intention (PI) PL choice	SIP → PL purchase (+) SIP → PI (+) PI → PL choice (+) PL product SPI → PI (+) PL product PV → PL choice (–) PL product PV → A (+) A → PL choice PI → PL choice
Zielke and Komor (2015) [75]	1. Price consciousness: value consciousness, price–quality schema, prestige sensitivity, preference toward Ps and discounter preference Hypermarket preference 2. Preference toward PLs: discounter preference and hypermarket preference	The negative role (price and value consciousness) increases preferences for PLs, discounters, and hypermarkets (+) The positive role (price–quality schema, prestige sensitivity) decreases preferences for PL products and discounters but increases preferences for hypermarkets in low-price categories (+)
Fall-Diallo et al. (2015) [76]	Butter (3 types of PLs: standard (S), organic (O), local (L)) Yogurt (3 types of PLs: S, O, L)	-
Delgado- Ballester et al. (2014) [77]	Store image (SI) Functional risk (FR) Financial risk (FiR) Social risk (SR) Psychological risk (PR) Price unfairness (PU) Value consciousness (VC) Consumer perceptions (CP)	+ SI reduces CP of the FR and FiR of PLs to a greater (lesser) degree with diminishing (rising) levels of VC (+) + SI reduces CP of the SR of PLs to a greater (lesser) degree with rising (diminishing) levels of consumer VC (–) + SI increases CP of the PR of PLs to a greater (lesser) degree with diminishing (rising) levels of consumer VC (–) Perceptions of FR, FiR, SR, and PR associated with PLs diminish the perception of the price unfairness of an alternative manufacturer’s brand (+)
Bauer et al. (2013) [29]	Study 1: main purchasing motives Study 2: (a) Experiment: 6 groups of PL products: local, global, or organic cereal products and nonorganic cereal products (b) Purchasing motives: Healthiness (PH) Hedonism (PHe) Environmental friendliness (EF) Food safety (FS) Study 3: the same 6 groups of products: Purchase intention (PI) Price premium (willingness to pay price premium) (WP)	Organic label (OL) of global (G)/local (L)/PLs causes a higher degree of PH than the respective G/L/PL brand without an OL (+) OL of G/L/PLs causes a higher degree of PHe than the respective G/L/PLs without OL (+) OL of G/L/PLs causes a higher degree of perceived EF than the respective G/L/PLs without OL (+) OL of G/L/PLs causes a higher degree of perceived FS than the respective G/L/PLs without an OL (+) OL of G/L/PL products leads to a higher PI than the respective G/L/PL products without an OL (+) OL of G/L/PLs leads to a higher WP a price premium than the respective G/L/PLs without an OL (+)
Fall Diallo et al. (2013) [78]	Store image perceptions (SIP) PL price image (PI) Value consciousness (VC) Attitude toward PLs (A) PL purchase intention (PIn) PL choice	SIP → PIn (+) SIP → PI (+) PIn → PL choice (+) SIP → PI (+) PI → PIn (+) PL product PI → PL choice (+) PI → PIn → PL choice (+) VC → PIn (+) VC → PL choice (+) VC → A (+) PIn → PL choice (+) VC → A (+) A → PL choice (+) PIn → PL choice (+)
Herstein et al. (2012) [79]	Choice of 2 types of brands (NB and PL), 5 food and 5 nonfood products Brand dimensions: brand name, packaging, country of origin Individualism (I): vertical (VI) and horizontal (HI) individualism Measure of materialism (M) Need for cognition (NC)	I is correlated with the inclination to purchase PLs M is correlated with the inclination to purchase PLs The need for cognition is correlated with the inclination to purchase PLs There will be cross-cultural differences in the inclination to purchase PLs Culture moderates the effect of personality on preference for PLs vs. NBs
Wyma et al. (2012) [80]	Brand preference (25 products available in NB and PLs) Psychographic statements related to brands Demographic characteristics	-
Tifferet and Herstein (2010) [81]	Willingness to purchase (NB or PL) for 10 types of products (5 food products and 5 nonfood products) Brand image, 3 factors: importance of packaging design, manufacturer’s brand name reputation, and country of origin Individualism and collectivism	Does individualism affect consumers’ preference for PLs vs. NBs? Do consumers with high levels of individualism show a lower inclination to purchase PLs? Does individualism affect consumers’ perceived importance of brand image dimensions? Do consumers with high levels of individualism attribute greater importance to brand image dimensions, such as packaging design, country of origin, and PL reputation? Are there cross-cultural differences within a specific country, namely, Israel?
Glynn and Chen (2009) [82]	1. Factors: Purchase mistake (PM) Quality variability (QV) Search vs. experience (S vs. E) Price consciousness (PC) Price–quality perception (PQP) Brand loyalty (BL) PL purchase 2. Average scores by PL product category (factors as above): canned fruit, toilet tissue, fresh milk, cheese, fruit juice, potato chips, biscuits, bread breakfast cereal, pet food	Are consumers more likely to buy PLs where they perceive lower consequences of making a mistake in brand selection (–)/variability in quality between brands (–)? Is it possible to accurately assess product quality of important attributes and benefits based on written descriptions only (–)/are consumers more price-conscious (+)? Consumers are less likely to buy Ps if they have an elevated perception of quality relative to price (+) Brand loyalty reduces consumers’ propensity to buy PLs (+) Consumers’ propensity to buy PL products is determined by gender/age (–) Consumers are less likely to buy PLs if they have more household income/formal education qualifications (+) Large households are more likely to buy PLs (+) Purchase of PLs is moderated by differences in PL category share (–)
Anchor and Kourilová (2009) [83]	Study 1: Importance of price Importance of quality Importance of confidence Study 2: perception of the Tesco PL category: *Tesco Value*, *Tesco Standard* Purchase frequency (PF) Perceived quality (PQ) Perceived price (PP) Confidence (C)	In both countries, the Tesco brands have the same PF (–) In both countries, the PQ of the Tesco brands is of the same level (–) In both countries, the PP of the Tesco brands is of the same level (–) In both countries, the C in the Tesco brands is of the same level (–) In both countries, a significant relationship between gender and perception of measured characteristics exists (–) In both countries, a significant relationship between age and perception of measured characteristics exists (–) In both countries, a significant relationship between income and perception of measured characteristics exists (+) In both countries, a significant relationship between purchase frequency and perception of measured characteristics exists (0)
Kara et al. (2009) [84]	Perceptions about manufacturers vs. PLs: budget conscious, value conscious, price conscious, discount conscious Consumer’s previous experience, sensory perception Content perception, PL purchase/use	Consumers’ consciousness (+) → PL perceptions (+) Consumers’ previous experience (+) → PL perceptions (+) Consumers’ consciousness (+) → consumers’ previous experience (+) BS perceptions (+) → PL purchase/use (+)
Albayrak and Aslan (2009) [85]	Brand preferences: NB food product preference analysis of NB food consumers PL food product preference analysis of NB food consumers PL food product preference analysis of PL food product consumers NB food product preference analysis of PL food product consumers	-
Cheng et al. (2007) [86]	2 categories of products for NB: international PL (IPL), and local PL (LPL) Perceived quality (PQ) Brand leadership (BL) Price perception (PP) Brand personality (BP)	The quality of NB products is perceived to be superior to that of IPL products, while the quality of IPL products is perceived to be superior to that of LPL products (+) Consumers perceive the price of NB products to be being significantly higher than IPL products, and the price of IPL products to be higher than LPL products (+) Consumers count on NBs for better brand leadership, on IPLs for worse brand leadership, and LPLs for nonbrand leadership (+) Consumers perceive the brand personality of NBs to be significantly superior to IPLs, and the brand personality of IPLs to be superior to local PLs (+) Product categories moderate the interaction of PQ (–)/PP (–)/BL (–)/BP (+) across NBs, IPLs, and LPLs
Mieres et al. (2006) [87]	A. Difference in perceived risk between PLs and NBs Perceived quality of PLs/NBs (PQ) Reliance on the extrinsic attributes of a product (REA) Specific self-confidence (SSC) Familiarity with PLs (FPL) Experience with product category (EPC) B. Perceived risk (PR): Functional risk (FR) Financial risk (FiR) Social risk (SR) Physical risk (PR) Psychological risk (PsR) Time risk (TR)	PO → Difference in PR (–) REA → Difference in PR (+) REA → PQ (+) SSC → Difference in PR (–) SSC → REA (–) FPL → REA (–) FPL → PQ (+) EPC → Difference in PR (–) EPC → SSC (+) EPC → REA (–) EPC → FPL (+) EPC → PQ (+)
Akbay and Jones (2005) [88]	A. Lower-income consumers 1. PB share/NB share 2. PB price/NB price B. Higher-income consumers 1. PB share/NB share 2. PB price/NB price C. Demand equations of 9 food categories for PLs and NBs in lower- and higher-income areas D. Demand elasticities for 9 food product categories for PLs and NBs in lower- and higher-income areas: Expenditure elasticity Price elasticity Promotion elasticity	-
Kurtulus et al. (2005) [89]	Price consciousness (PC) Financial constraints (FC) Quality consciousness (QC) Store loyalty (SL) Shopping mavenism (SM) Time limitation (TL) Brand loyalty (BL) Tendency to purchase PBs (T)	T → PC (+) T → FC (–) T → QC (–) T → SL (+) T → SM (+) T → TL (–) T → BL (–)
Semeijn et al. (2004) [90]	1. Store image (layout, merchandise, service) (SI) 2. Consumer attitude toward PLs (CA) (a) Perceived overall quality of PLs (PQ) (b) Likelihood of purchasing PLs (LP) 3. Risk factors: functional (FuR), psychosocial (PR) and financial (FR)	A positive relationship exists between perceived SI and CA (+) CA is inversely related to FuR associated with the perceived difficulty for the retailer to produce that product (+) The effect of SI on consumer attitude toward PLs is mediated by FuR associated with the perceived difficulty for the retailer to produce that product (+) CA is inversely related to the perceived PR associated with the usage of the product (+) The relationship between SI and CA is mediated by PR of usage (+) CA is inversely related to perceived FR associated with quality variance in the product category (+) The relationship between SI and CA is mediated by the perceived FR of usage (–)
Veloutsou et al. (2004) [91]	1. Change of behavior toward PLs and supermarkets 2. Product attributes: (A) Brands (PLs and NBs) Perceived quality Value for money Appealing packaging Perceived taste (B) Brands (for PLs and NBs) Importance of price Quality Packaging Advertising Fulfillment of expectations (C) Country: factors same as in A point (D) Country: factors same as in B point 3. In-depth interviews with 5 consumers in each country	Consumers give similar emphasis to choice criteria when purchasing PL and NB products (–) Consumers evaluate PLs and NBs similarly (–) Greek (G) and Scottish (S) consumers have similar degree of familiarity with buying PLs (–) G and S consumers give similar emphasis (mental weighting) to choice criteria when purchasing PLs (–) G and S consumers evaluate the PLs (quality, value for money, appealing packaging, and taste) similarly (–) G and S consumers have similar readiness to purchase PLs (–) G and S consumers have similar readiness to change their behavior toward PLs (–) Habits toward the product category are influential on the willingness to buy PLs (+) PL choice criteria are influential on the willingness to buy PLs (+) Consumers’ demographic characteristics are influential on the willingness to buy PLs (+) Satisfaction with PLs from a certain supermarket will increase the consumers’ loyalty to that supermarket (+)
Miquel et al. (2002) [92]	PL product purchase: (1) Knowledge of the category (2) Perception of differences (3) Willingness to buy PL products	Greater knowledge of the category leads to prefer NBs (+) The greater the belief that differences exist between the different alternatives, the less likely the possibility of the individual buying PLs (–)
Vaidyanathan and Aggarwal (2000) [93]	Product attitude (PA) Quality perceptions (QP) Value perceptions (VP) Value consciousness (VC)	PA toward unfamiliar PL products with a familiar NB ingredient will be more favorable than that toward unfamiliar PL products with an unbranded ingredient (+) QP of unfamiliar PL products with a familiar NB ingredient will be more favorable than that of unfamiliar PL products with an unbranded ingredient (+) PA toward a familiar NB name (ingredient) will not be unfavorably affected by an association with an unfamiliar PL product (+) QP of a familiar NB name (ingredient) will not be unfavorably affected by an association with an unfamiliar PL product (+)

**Table 4:** General findings and managerial implications for the studies included in the systematic survey.

Author, Year	Key Findings	Practical Implications
Temmeman et al. (2021) [54]	Products were identified to be healthier with Nutri-Score (NS), but the healthiness of products ranked in 5 categories was evaluated significantly differently. Purchase intentions were higher for products with positive NS than for products with negative NS. Due to the increase in the quality of PL products, consumers accept and trust PL products, and are therefore more loyal to them, regardless of NS.	The NS system should be introduced on the European nutrition label and is an effective option to manage the growing obesity epidemic.
Kadekova et al. (2020) [55]	PL products were perceived to be products of good and adequate quality, available at a reasonable price. PL product categories, such as milk and dairy products, mineral water, lemonade, and juice, were the most frequently purchased, while alcoholic beverages and frozen semifinished products were the least frequently purchased. The purchase of PL products is influenced by traditional forms of marketing communication, recommendations, and provision of free samples for tasting, and some form of promotion.	Packaging can influence consumers’ decisions, which retailers and producers should take advantage of. The boundaries between traditional and PL products are gradually blurring, and the possibilities to increase the attractiveness of PL yogurts can be based on increasing Slovak consumers’ awareness of PL products and their manufacturers.
Czeczotko et al. (2020) [43]	In Poland, dairy products, cereals, and nonalcoholic beverages under PLs were the most frequently purchased, while in the UK, bread, dairy products, fruit, vegetables, and frozen products under PLs were the most frequently purchased. In both countries, consumers were least likely to buy PL alcohol. The ability to buy the same product repeatedly and the availability of PL products were the most important factors in the choice of PL products. Consumers are positive about the current development of PL products pointing to a better visual presentation of PL products, lower price, and overall improvement in the quality of PLs.	The results are crucial for retail companies and international chains to identify the conditions for the development of PL products toward sustainable products and to identify tools to develop products with sustainability-based competitive advantage in the dynamically changing retail market.
Anitha and Krishnan [57]	Consumers make impulsive purchases, especially when they observe any discounts and offers or are given free products of premium PLs. The individual level of income plays an important role in consumers’ impulsive buying behavior.	The promotion of impulse buying must closely match consumer choice and preference and situational factors. Researchers can continue the study with internal and external factors, together with promotional techniques and the role of brands.
Košičiarová et al. (2020) [58]	The boundaries between traditional and PL products are gradually blurring, and customers are beginning to realize that PL products are a suitable alternative. The possibilities of increasing the attractiveness of PL yogurts could be based on raising awareness about PL products among consumers. Consumers still hesitate to buy PL products because they have no experience with these products and do not know their producers.	The results can be used as a guide to increase the attractiveness of yogurts and, thus, its consumption by consumers. This research can serve as a tool to raise awareness among both professionals and the public about the existence of PLs, their importance, and their advantages and potential disadvantages.
Singh and Singhal (2020) [59]	Consumers consider PL products to be low-quality products compared to products of producer brands, but the PL product quality varies among different product categories The key to ensuring the good quality of PL products is to build brand equity and offer products at a premium price.	PLs should be differentiated by spending more on advertising, promotion, and internal and external communication, as the third-generation PLs build loyalty to the store or to the chain. Retailers should produce high-quality products that help them to build loyalty toward the store chain, thus creating good brand and store image. PL products should stand out in future; therefore, retailers should continuously understand consumers and come up with innovative products that will compete with branded products.
Košičiarová et al. (2020) [56]	PL products are perceived to be products of good quality, and PLs are associated with products of adequate quality at a reasonable price. The most frequently purchased product categories available under PLs are milk and dairy products, meat and fish, which are purchased every week, snacks and mineral water, lemonades, and juices, which consumers buy once a month or once a week. Packaging can influence consumers’ decisions, and plays an important role in their purchasing decisions and product evaluation.	Traditional forms of marketing communication, such as word of mouth marketing and friends’ recommendations, and the provision of free samples for tasting are more preferred by consumers and should therefore be used by retailers and manufacturers.
Prediger et al. (2019) [60]	Shorter flyers had a stronger influence on consumer intentions to purchase PLs, especially in the yogurt category. Including a wide variety of products under PLs on the flyer is more effective in increasing store traffic and sales.	The features of the flyer, i.e., number of brands and proportion of content, can be used to modify consumer perceptions of variety and store image.
Gómez-Suárez et al. (2019) [61]	Smart shoppers’ self-concept influenced their attitude toward PLs and NBs. There is a positive and significant causal relationship between the smart shopper self-concept and the attitude toward promoted NBs. The smart shopper self-concept was significantly and positively correlated with attitudes of NBs in all countries, except the UK. Spain and Germany were the only countries that showed a significant correlation between the smart shopper self-concept and the attitude toward PLs.	Using a more complex shopping basket, researchers may better understand how various degrees of perceived risk (whether economic, functional, or social) affect the relationship between the smart shopping mechanism and brand attitudes. From a managerial standpoint, the results can assist international marketing practitioners in developing strategies to target smart shoppers. Some degree of standardization in segmentation, positioning, and communication strategies should be relied upon.
Salazar-Ordóñez et al. (2018) [62]	People with positive perceptions of PL value show positive attitudes toward refined olive oil (ROO) only. Consumers may associate PLs of olive oil with ROO rather than extra-virgin olive oil, and PL perceived value may reinforce ROO features.	The results indicate the role of feelings aroused by the potential anticipated consequences of product use, healthy lifestyles, shopping habits, the perceived value of PLs, and perceived taste. The perceived value of PLs determines the formation of attitudes toward ROO. It is fundamental for small and medium enterprises to undertake effective marketing strategies to highlight the added value of their products.
Liu et al. (2018) [63]	Higher levels of BESC (brand engagement in the self-concept) resulted in greater purchases of NB products, and BESC affects consumer preferences for broad brand categories (such as NBs or PLs). Decreased preference for NBs (compared to PLs) suggests that the importance of brand self may decrease when consumers (with higher BESC disposition or presenting brand engagement manipulation) experience a self-concept threat unrelated to brand self.	NBs should highlight aspects such as quality and taste in their marketing efforts; such attributes are unlikely to activate the overall self-concept of highly branded consumers and, thus, negatively affect preferences for NBs over PLs. NBs and PLs competing for the same consumer groups can benefit from understanding and carefully considering the interaction of how people perceive themselves and their branded self in developing marketing strategies for their respective target markets.
Valaskova et al. (2018) [16]	Regardless of respondents’ demographics, consumers purchase all categories of PL products, with dairy, durable goods, and paper hygiene being the most preferred. For each PL product category, consumers identify key factors that drive them to purchase. Price is the most important factor considered when purchasing dairy products, quality in the case of hygiene products, product composition for detergents, advantageous packaging for animal food, packaging and design for frozen goods, and the range of products for cosmetics. Customers who are more likely to purchase PL products identify the reasons for their purchase as cost effectiveness, quality, and loyalty to the retailer.	PLs should increase the range of products available, and thus intensify inter-brand and price competition. PLs should change the relationship between retailers and their suppliers. Consumers perceive PL products much more positively compared to the past, when PL products were perceived as low-priced products of inferior quality. Therefore, PLs should now aim at increasing market shares and introducing new product categories.
Vázquez-Casielles and Cachero-Martinez (2018) [64]	The introduction of economy (EPLs) or premium PLs (PPLs) increases “quality variation” within the PL brands. The introduction of top-quality PPLs can adversely affect customer trust. The introduction of EPLs may be beneficial for the second-tier NBs because the retailer’s assortment includes average options in the quality dimension. The introduction of PPLs decreases the probability of choosing EPLs and standard PLs much more than the probability of choosing premium products. When EPLs and PPLs are introduced, the probability of choosing standard PLs decreases, especially for high-volume shoppers and PL-loyal customers. Loyal PL customers and high-volume shoppers are more likely to appreciate the introduction of EPLs and PPLs.	Retailers can position EPLs as discount brands, creating stand-alone brand names (i.e., pseudo-brands) instead of retailer brands (umbrella brands), and by using other prominent shelf areas by displaying only discount products. The retailer can compete by introducing PPLs that offer the customer new products, experiences, and concepts that NBs do not offer. If retailers can produce PPL products that are something different, unique, or new in the category, they will gain greater market share and better results. The threat of introducing PLs can be used to negotiate better retail margins with second-tier and premium-quality NBs.
Garczarek-Bąk (2018) [65]	Consumers are influenced by many factors when choosing PL products. Service quality rating is a statistically significant differentiating variable between men and women for only one retailer. Knowledge of the price of PL products does not influence the decision to purchase PL products. The relatively higher left frontal activation (i.e., higher approach motivation) during the pre-decision period in some cases predicted a purchase decision.	Retailers should analyze the determinants of PL product selection in detail because the eye tracking study did not reveal differences between women’s and men’s esthetic sensitivity toward the presented PL products.
Meliana (2018) [66]	The price of PL products is lower and more reasonable compared to similar products of manufacturer brands. Strategies of locating the minimarket close to houses promote shopping interest in older consumers.	For PL products, quality should be analyzed in conjunction with price because the index of customer confidence in the quality of PL products has a high value.
Modica et al. (2018) [67]	The comparison of products of major brands and PLs showed higher positive rating values for the products of major brands than for the PL products belonging to the comfort food category. A higher purchasing tendency has been found toward foreign food products in comparison with local food products during the visual and tactile exploration phase. Higher mental effort occurs when interacting with foreign products during the visual exploration phase and the visual and tactile exploration phases.	The results could deepen the knowledge on the neurophysiological response to food products characterized by different natures in terms of hedonic value familiarity.
Schouteten et al. (2017) [68]	Research setting and brand information may, under certain conditions, influence the sensory and emotional profiles of food products. Information such as brand, content information, health information, and package could alter sensory perception.	Scientists and food companies should consider the impact of the chosen methodology on organic validity when conducting sensory testing with consumers, as the laboratory context may lead to a more positive evaluation compared with a home-use test.
Jara et al. (2017) [69]	Economic PLs (EPLs) build their equity with reinforced packaging, and organic PLs (OPLs) maximize their brand equity by using simple packaging. EPLs do not create perceived value when these brands use simplified packaging. The type of packaging is a significant determinant for differentiating PL equity through its impact on perceived quality.	Firms can decide to remove secondary packaging from their OPLs. Retail managers should work effectively to develop perceived quality, particularly by aligning their packaging with store brand positioning. It would be important to expand product categories to better appreciate the impact of packaging on value created.
Gomez-Suarez et al. (2016) [70]	Price-driven consumers favor NBs over PLs. Customers perceive retail brands to be an alternative with a good price–quality balance. Quality based on brand image and reputation has a significant positive impact on attitude toward PL products. Impulsiveness has a slight positive impact on intention to purchase PL products.	Retail managers should continue to invest in producing innovative products and explore new ways of improving the overall shopping experience. Retailers could minimize perceived risk by offering product warranties, encouraging product trials, and implementing customer-friendly product return processes. Impulsiveness positively influences PL purchase intention, so retail managers can use packaging design, attractive point-of-sale promotions, and communication to encourage unplanned PL purchases.
Marques dos Santos et al. (2016) [71]	Some brain structures are more active/inactive for NBs than for PLs, both marked with real market prices. Price is a strong factor influencing purchase decisions. Brain activation/deactivation patterns suggest that accepted models of brain functioning are not adequate to explain brand decisions. There is an approach to understanding how such brand categories are perceived, revealing the neural origins of the associated psychological processes.	This study may be categorized as discovery research or pure research, aiming to contribute to the construction of a brain-based model of brand perception.
Thanasuta (2015) [72]	Price-conscious consumers are most likely to purchase PL products in low-differentiation categories.	PLs should maintain a low-price strategy while striving to continually improve quality to attract additional quality and value-conscious consumers. The ability to offer an acceptable-quality product at an affordable price will increase the opportunity for PLs to capture value-conscious consumers. When creating NBs, the focus should be on brand image.
Schnittka (2015) [38]	The price level of the grocery store moderates the effect of PL tiers on consumer preference for PLs. Premium PLs are more promising for high-priced grocery stores than for low-priced grocery stores and in high-brand-importance product categories, while economy PLs are more promising for low-brand-importance categories.	Premium PLs are more promising for low-priced grocery stores that offer discounts because they meet consumers’ primary shopping objective of purchasing products at low prices. Premium PLs in higher-priced grocery stores and supermarkets seem to be questionable, with lower profitability and potential negative side effects on the brand image of a particular grocery store due to inadequate offers.
Monnot et al. (2015) [73]	Eliminating overpackaging has a significant positive effect on perceived environmental friendliness and a significant negative effect on perceived convenience. Eliminating overpackaging has an influence on the image of mimic PL products, especially on perceived quality, convenience, and environmental friendliness, but no impact on generic PL products. Overpackaging can be legitimately eliminated without affecting the perceived quality of a product positioned as “economical,” while reducing the production costs of overpackaging for the retailer.	Communication campaigns focusing on the fact that the elimination of overpackaging does not affect product quality and emphasizes the benefits of the product attributes (convenience, price, environmental friendliness) are advisable. In the context of sustainability, retailers may present the elimination of overpackaging to reduce waste and as a possibility of selective waste collection.
Diallo et al. (2015) [74]	Consumers purchase Extra PLs not only for price image perceptions, but also because of attitudes toward PLs, while they purchase Carrefour PLs because of store image perceptions and attitude. Age and income are more strongly associated with buying Extra PLs, while gender is more strongly associated with buying Carrefour PLs. The Brazilian market shows some deviations from both developed and other emerging countries.	Extra retail managers should focus on the image of their stores, while Carrefour should pay attention to the price positioning of PLs. The Carrefour chain should focus on younger and less wealthy consumers, who constitute a huge segment in Brazil. Retail managers operating in Brazil should pay attention to attitudes toward PL products and purchase intention to increase individual customers’ product purchase choices and sales. Retail managers should focus on improving the perception of store image in an emerging country, such as Brazil, to increase sales of Ps.
Zielke and Komor (2015) [75]	Price–role orientations, store format, and PL preferences differ in high- and low-income countries, and low incomes increase price consciousness. Country and low income have a positive effect on discounting preferences, indicating that in emerging countries, low-income groups have stronger discounting preferences compared to high-income groups. German customers have at least marginally higher preferences for PLs and discounters in low-price functional categories because they are as price- and value-conscious as Polish customers. Price–quality inferences and prestige sensitivity are less important.	A “soft discount” concept with a higher share of NBs may be more appropriate for emerging markets than a “hard discount” concept. Retailers should adapt the strategic positioning of store formats in emerging countries, considering cross-national differences in price–role orientations. Hypermarkets currently do not adequately address the higher positive role of the high-priced category in the emerging market analyzed, although the preference for hypermarkets is higher than in a developed country. Emerging country retailers can compete with international retailers by more effectively incorporating price–role orientation through store formats, but with economic development, price–role orientation and preferences may change and become more like those in developed markets.
Fall-Diallo et al. (2015) [76]	The buying behavior toward PLs depends not only on the macroeconomic situation and the product category, but also on PL variety. Most established relationships between P buying behavior and its antecedents differ when the macroeconomic situation changes (from expansion to a crisis).	Retailers should no longer manage PL products as a homogeneous range of products. The macroeconomic situation should be carefully monitored based on product category characteristics. Retailers should more closely monitor consumers’ prior experience with PLs, as this explains the buying behavior of PL consumers in both expansion and crisis periods for low- and high-frequency categories.
Delgado-Ballester et al. (2014) [77]	Store image has different effects on four categories of perceived risk, the strength of which varies with value consciousness. Perceptions of price unfairness with manufacturer brands are attenuated by the financial and functional risk of buying PLs, but increased by social and psychological risk. Price differences are interpreted in terms of quality differences, as consumers frequently assume that price and quality are highly correlated: “you get what you pay for.”	For retailers, the key implications concern the awareness and management of customer perceptions of relative risks, and the impact of value consciousness on the use of store image as a heuristic decision-making cue. The retailers need to invest in the creation and maintenance of a positive store image in consumers’ minds, as it has a significant impact on reducing the perceived risk associated with PLs. For manufacturers, it is a necessity to demonstrate clear product differentiation as a justification for higher prices.
Bauer et al. (2013) [29]	In the conventional food range, PLs are perceived to be less healthy, less hedonic, less environmentally friendly, and less safe compared to a local and global brand, and are characterized by a lower price premium and purchase intention. Certified organic PLs are perceived to be almost as healthy, hedonic, environmentally friendly, and safe compared to local and global brands and are characterized by the same price premium and purchase intention. Brand is more important than label, and manufacturers’ brands are the most effective in profiting from the use of organic labels.	Brand owners must ensure that the organic label is consistently communicated, which can be used to develop more organic food product lines. The owners of strong brands need to assess whether organic labeling might erode the brand value of the established products or cause the value to stagnate. The use of organic certification is primarily suitable to PLs, which would benefit the most from the effect of the organic label.
Fall Diallo et al. (2013) [78]	Perception of store image, price image of PLs, value consciousness, and attitude have a significant and positive influence on the purchase behavior of PLs. The indirect effect of perceived store image on store brand choice confirmed that consumers use store image, including service, layout, and merchandise, as heuristics to infer the quality of PL products before choosing.	Retail managers must offer PLs that attract consumers not only in terms of price and quality, but they must place greater emphasis on both price image and store, as these factors influence consumer purchase behavior. For retail managers, these results may mean that PLs are becoming increasingly popular among more groups of consumers, including those with high household incomes. Retailers would benefit if they offer higher value-added products (i.e., premium products) to attract and retain customers loyal to PLs.
Herstein et al. (2012) [79]	Individualism and materialism influence the perceived importance of brand dimensions.	International retail chains should identify the profile of specific markets or closely related markets and develop internationalization and localization marketing strategies. Retailers should emphasize the extrinsic characteristics of their PLs, such as packaging design, country of origin, and the brand name.
Wyma et al. (2012) [80]	Consumer choice of PL products is associated with the product category. Brand preference depends on the demographics for each product, and psychographic factors are not significant in terms of product choice. Consumers are not well informed about PL products in general. The image of PL products may be at risk due to the tendency to associate cheaper products with lower quality.	Retailers and manufacturers should determine the demographic and psychographic profile of the product-specific target market when producing or marketing PL products. A broader survey, covering a wider range of products, should be conducted with a representative sample to understand the reasons for consumers’ brand preferences. The types of PL products offered in the market need to be revised, as not all products appear to be equally viable.
Tifferet and Herstein (2010) [81]	Individualistic consumers are less likely to purchase PLs. Cultural groups show marked differences in the importance they attribute to the country of origin. Consumers from immigrant cultures placed more importance on the country of origin of PL products compared to Hebrew-speaking consumers.	Marketers who deal with PLs should invest less in marketing their products to individualist consumers because they are less likely to purchase PLs. Local distributors should not invest heavily in creating different branding strategies for the four subgroups of consumers. Marketers should focus their branding strategy on a common marketing concept that reflects the country’s values.
Glynn and Chen (2009) [82]	Quality variability, price consciousness, price–quality association, and brand loyalty influence consumers’ willingness to buy PL products. The impact of price consciousness and quality variability on PL product purchasing depend on product category and PL market share. For retailers, PL value is less important in some categories.	Retailers should pay particular attention to maintaining and improving the quality of their PL products, attempting to increase PL share by improving the ingredient quality as well as the packaging, design, and labeling. Retailers can face competition from branded manufacturers by targeting PL consumers from different demographic groups. Producers should emphasize price–quality aspects in their marketing communications because the relationship between price and quality has a positive impact on the performance of NBs.
Anchor and Kourilová (2009) [83]	The general opinion of Tesco’s PLs is slightly less positive among Czech than British customers. Czechs buy more standard products, while the British slightly prefer the value brands. In both countries, the quality of Tesco products is perceived to be better than other brands. Tesco’s PLs enjoy a higher level of trust than other established brands.	Tesco needs to adjust its branding strategies and facilitate full penetration of its brands into all product categories. The results of the research can help Tesco in its expansion in Central and Eastern Europe in general and with its branding.
Kara et al. (2009) [84]	Consumers’ perceptions of PLs were significantly influenced by their levels of consciousness as well as previous experiences. Consumer consciousness positively influences perceptions of PLs and, subsequently, their purchase of the brand. Consumers use the brand name primarily as a cue to judge the quality of the product.	Retailers should continuously focus their efforts on trying to create a strong brand image for their PLs. It is important to invest in promotional campaigns to familiarize consumers with their brands and encourage them to make their first purchase. Consumers’ consciousness contributes to a positive perception of PLs and, therefore, marketing strategies should be designed to emphasize the “value” aspects of the offering. Effective advertising and promotion should position these products as products of very high quality and value, and as accessible ones.
Albayrak and Aslan (2009) [85]	Consumers of manufacturer brand products place more importance on brand and quality, while PL consumers are more price sensitive and more open to trying new brands.	Retailers should use strategies other than simply maintaining low prices and making products available to encourage customers to buy PL products. PL products become as attractive as manufacturer brand products when effective marketing communication, adequate packaging, and product diversity are offered to consumers.
Cheng et al. (2007) [86]	Consumers perceived brand types differently, meaning that NBs were perceived to be significantly better than international PLs, while international PLs were perceived to be better than local PLs based on all attributes except price. For international and local PLs, product imitation strategies are used. There is no difference in price perception between NBs and international PLs.	It is important for international PL managers to emphasize that purchasing high-quality and innovatively labeled products is associated with value for money. Managers of international PLs should be cautious in applying pricing strategy across different types of product categories.
Mieres et al. (2006) [87]	Relying on the external attributes of a product to evaluate its quality, such as brand name and price, is a key element for a consumer to make a purchase decision for NBs vs. PLs. Consumers are becoming more conscious of the consequences of their purchasing decisions, beginning to associate greater risk with their purchases, and trusting NBs more.	Retailers need to keep in mind that PLs are still seen as an inferior alternative to NBs, and are considered to pose a greater purchasing risk. Retailers need to explain to consumers that the lower prices of their brands are not a consequence of inferior quality, but rather are the result of major cost savings, for example in the way they are marketed. The development of commercial policies aimed at enhancing brand image or corporate identity can help to increase the familiarity with and prestige of PLs, and prevent them from being regarded as an alternative.
Akbay and Jones (2005) [88]	Higher-income consumers are more likely to purchase NB products and, therefore, manufacturers have often lowered prices to slow, or effectively manage, the penetration of PL products. Lower-income consumers are shown to perceive more easily the binding constraints of income and make purchase decisions to maximize their utility. Income plays a significant role in purchase decisions. PL products are strong substitutes for NB products, whereas NB products are weak substitutes for PL products.	n.a.
Kurtulus et al. (2005) [89]	Price consciousness is the most effective driver of consumer preference for retail brands. Quality-conscious consumers, regardless of shopping mavenism and brand loyalty, attach importance to time constraints, which leads to store loyalty. Quality consciousness is strongly correlated with brand and store loyalty. People who have time constraints may show loyalty to stores that offer product variety and parking spaces, and which are close to where they live.	Retailers should consider these results when developing marketing strategies for their PLs. Retailers should consider consumers’ price sensitivity in their price promotions and pricing policies to increase the effectiveness and efficiency of marketing activities. It is worthwhile to analyze the impact of consumers’ psychographic factors on their willingness to purchase PL products by including evenly distributed samples (e.g., gender, education, income).
Semeijn et al. (2004) [90]	The appeal of manufacturers’ brands may be waning as consumers become well informed about commodity products. Developing, nourishing, and sustaining store image can create opportunities for differentiation and positioning relative to other chains, and lead to profitable PL sales. Differences in perceived store image are a consequence of variation in retail strategy, store design, and commitment to meeting customer needs.	Retailers should take the lead in the further development of PLs. New PL products may have greatest potential in low-risk product categories. Retailers should, therefore, focus on aspects such as store environment, merchandise quality and value, and customer service.
Veloutsou et al. (2004) [91]	Price and packaging are more considered when buying NB products, while PL products are perceived to be high-quality products. Greeks’ and Scots’ experiences with PLs, the selection criteria they use, and their views on PLs are different. Greeks are less familiar with PLs, consider communication and impulse factors more when purchasing PL products, and are less willing to buy PL products than Scots. The customers who are satisfied with PL products are more loyal to a certain supermarket, so the PL range should be carefully managed.	Retailers who want to introduce and support PL products in the European Union over the long term must remember certain regional differences, as customers living in different regions have different experiences with and expectations from PL products. The increasing recognition of brands as sources of sustained competitive advantage highlights the importance of the assumptions and models underlying the brand strategies used by organizations. Constant market monitoring is a prerequisite for the success of production and retail brands.
Miquel et al. (2002) [92]	The greater the knowledge the consumer possesses of the product category being evaluated, the greater the possibility that the PL products will be preferred. Perceived differences between the two brands are in favor of the NB products and against buying PL products. The level of involvement depends on the consumer rather than the product and situational factors that may be present at the time of the purchase decision. Consumer knowledge of the product category and perceptions of differences between NB products and PL products influence purchase decisions.	If manufacturers are to maintain their position as leaders, they need to know that distribution companies are devoting increasing resources, time, and effort to developing and promoting their PLs. Trust, placed in the store and the brands, can be turned into a distributor’s competitive advantage, not only in competing with manufacturers, but also in competing with those distributors who also offer their PLs.
Vaidyanthan and Aggarwal (2000) [93]	The association of branded ingredients with PL products can have a positive impact on consumers’ evaluation of an unfamiliar product.	Product partnerships between PLs and NBs have potential benefits and future profits.

## Data Availability

Data are available at the Department of Food Market and Consumption research in the Institute of Human Nutrition Sciences, Warsaw University of Life Sciences, in Poland.

## Appendix A

**Table A1:** Objectives and measurement items of studies included in the SLR.

Author, Year	Objective	Measurement Items
Temmerman, et al. (2021) [54]	To analyze the impact of the presence of the Nutri-Score and its five categories on consumers’ perceived healthiness perceptions and purchase intention. To analyze the impact of the Nutri-Score on perceived quality, perceived healthiness, and purchase intentions (national brands vs. PLs).	Study 1: 6 items in a 7-point semantic differential (SD) scale: PQ: 1 item; PH: 5 items 9 items on a 7-point Likert scale: PT: 5 items, PI: 4 items Study 2: 4 items on a 7-point SD scale: PH: 1 item; FNS: 3 items 20 items on a 7-point Likert scale: PI: 4 items; NK: 8 items; PhF: 5 items; Db: 3 items
Kadekova, et al. (2020) [55]	To analyze the impact of packaging on consumer purchasing decisions in the yoghurt segment.	Questionnaire: 17 items, scale of 1 to 5 Blind test: on a scale of 1 to 5, with 1 being the best rating and 5 the worst The first test: tasting yoghurts without knowing it The second test: already-known packaging
Czeczotko, et al. (2020) [43]	To analyze the behavior of British and Polish consumers towards PL products, i.e., the frequency of purchasing PLs, the motives for purchasing products offered under PLs, the consumers’ opinions on PL development, and the length of the period of purchasing PL products.	36 items: PP: 5 items (single answer) FP: 8 items (5-point Likert scale) OCD: 6 items (5-point Likert scale) FPC: 10 items (5-point scale) SPL: 7 items (% scale)
Anitha and Krishnan (2020) [57]	To examine the impulse purchase behavior of PL products in modern retail outlets and the major factors influencing it.	26 items, 5-point Likert scale
Košičiarová, et al. (2020) [58]	To analyze customer preferences in the context of loyalty to the brand of selected food products in the segment of yoghurts.	Questionnaire: 10 items (5-point Likert scale) Blind test: on a scale of 1 to 5, with 1 being the best rating and 5 the worst
Singh and Singhal (2020) [59]	To understand consumers’ attitudes and preferences, as well as behavior, focusing on 3 types of PLs. To investigate how the grocery retailers are motivated to market the PLs.	23 items (5-point Likert scale)
Košičiarová, et al. (2020) [56]	To analyze the influence of packaging and marketing communication tools on consumer purchasing decisions in the dairy segment.	Questionnaire: 10 items (5-point Likert scale), Blind test: on a scale of 1 to 5, with 1 being the best and 5 the worst: -1st round–-5 items: color, flavor, fragrance, consistency, and the chocolate ratio -2nd round—7 items: color, flavor, fragrance, consistency, chocolate ratio, the attractiveness of the packaging, and grammage
Prediger, et al. (2019) [60]	To explain how store flyer features affect the store traffic and the consumers’ intentions to buy PLs. To analyze the moderating effect of consumers’ perceptions on the retailer’s assortment and the store.	Experiment: Factor 1: brand promoted on the cover page (+1 = NB, or −1 = PL) Factor 2: the page length of the store flyer (+1=20 pages, or −1=8 p.) Factor 3: use of an institutional slogan on the cover page (+1 = presence or −1 = absence) Online survey: 2 items (7-point Likert scale)
Gómez-Suárez, et al. (2019) [61]	To find out the extent to which smart shopping and its effect on consumer attitudes towards PLs and national brands is influenced by consumers’ cultural values.	Study 1: 18 items on a 9-point Likert scale—“guiding principle of my life” Study 2: 18 items on a 7-point Likert scale—smart shopper concept, attitude
Salazar-Ordóñez et al. (2018) [62]	To examine value for consumers of own-label or PLs.	7-point Likert scale for 13 items: AE: 4 items; AR: 4 items; PV: 5 items
Liu et al. (2018) [63]	To examine consumers’ preference for national brands and PLs and their tendency to include brands as part of their self-concept.	Study 1: 12 items (7-point Likert scale) Study 2: 7-point scale Study 3: 3 items on an SD 7-point scale
Valaskova et al. (2018) [16]	To determine factors and variables that significantly influence and shape the consumer’s perception and attitude towards the purchase of PL products.	6 items: 5-point Likert scale: choice from 10 categories of PLs
Vázquez- Casielles and Cachero-Martinez (2018) [64]	To analyze how the introduction of economy and premium PLs affects national brands and standard PLs for different customer segments.	18 items: 5-point Likert scale
Garczarek-Bąk (2018) [65]	To investigate the factors affecting PL products’ possible purchase decisions for different retailers. To analyze how motivation, measured by total fixation duration using EEG asymmetry over the frontal hemisphere of the brain, predicts PL purchase.	PPE: 6-point scale, from 1 (poor) to 6 (high) PI: The Juster scale, from 0 (not at all) to 11 (for sure) QA: 8 items on a 6-point scale
Meliana (2018) [66]	To explain how PLs can create an attractive store image and become a shopping preference for consumers.	8 items (5-point Likert scale)
Modica et al. (2018) [67]	To investigate the reactions of the EEG and the autonomic activities, as elicited by the cross-sensory interaction (sight and touch) across several different products. To investigate whether the brand (major brand or PL), familiarity (foreign or local brand), and hedonic value of products (comfort food or daily food) influence the reaction during their interaction with the products.	Each phase with eyes closed for 15 s and rating on the scale from −5 to +5: Experiment 1: VE, VTE; Experiment 2: TE, VE, VTE
Schouteten et al. (2017) [68]	To analyze the role of the research setting and brand information on the overall acceptance and sensory and emotional profiling of 5 strawberry yogurts.	1. Emotional profiling—18 emotional terms: -8 positive terms (contented, friendly, good, happy, interested, pleasant, surprised, satisfied) -8 negative terms (bored, disappointed, discontented, disgust, dissatisfied, frustrated, stressed) -2 neutral terms (calm, steady) 2. Overall liking: 5-point scale (from 1 = slightly to 5 = extremely) 3. Sensory profiling: 12 sensory terms (aftertaste, creamy, dark color, firm, fruity, milky flavor, sour, liquid, homogeneous, smooth, sweet, and thick)
Jara et al. (2017) [69]	To analyze PL equity by considering two PL’s positioning strategies: those with high perceived added value (the organic store brands), as opposed to economic brands.	11 items (5-point Likert scale) Respondents to look at an A3-sized image of a pack of four
Gomez-Suarez et al. (2016) [70]	To analyze the relationships between the different phases of the evaluation of PLs (attitude, preference, and purchase intention) in an international context.	1 item: scale (0 = NB and 1 = SB) 8 items: 7-point Likert scale
Marques dos Santos et al. (2016) [71]	To explore brain-based differences in perception of national brands and PLs. To study the influence of price as a differentiating characteristic of national brands and PLs.	15 explanatory variables (EVs): -12 items: type of brand (national and PLs), exhibited price (real market price and manipulated price), and the stage in the stimulus sequence (product, price, and decision) - 3 items: product, price, and decision for the overseas branded products
Thanasuta (2015) [72]	To investigate the relationship between consumer decision-making styles and actual purchases of PL products, using price consciousness, quality consciousness, brand consciousness, value consciousness, and risk perception.	7-point Likert scale for 23 items: PLs purchase: 1 item; QC: 4 items QC: 4 items; BC: 4 items; VC: 6 items; RP: 4 items
Schnittka (2015) [38]	To identify the moderating impact of the store, category, and PL characteristics on consumers’ preferences for premium vs. economy PLs.	7-point Likert scale: Study 1: 2 items Study 2a: 9 items Study 2b: 9 items
Monnot et al. (2015) [73]	To examine how eliminating overpackaging influences consumers’ perception of products sold under generic and mimic PL and purchase intention.	1. 5-point Likert scale for 17 items: PS: 3 items; PQ: 3 items EC: 3 items; PE: 2 items; PI: 2 items; PEF: 2 items; PC: 2 items 2. OP: 4 items (5-point Likert scale)
Diallo et al. (2015) [74]	To investigate the role of image and consumer factors in influencing the choice of PLs between two retail chains (Carrefour and Extra).	7-point Likert scale for 28 items: SIP: 9 items; SPI: 6 items; VP: 4 items A: 4 items; PI: 4 items; PL choice: 1 item
Zielke and Komor (2015) [75]	To extend cross-national research on price role orientations by focusing on culturally similar but economically different countries, relating differences to preferences for PLS and low-price store formats, and analyzing these effects for functional vs. hedonic and low- vs. high-price products.	1. 7 items (7-point Lichtenstein’s scale) 2. 12 items (7-point Lichtenstein’s scale scale)
Fall-Diallo et al. (2015) [76]	To investigate how previous experience with PLs and marketing policy variables affect PL purchasing behavior in two specific periods (expansion and crisis).	Variables to each product and period: price, feature, display, loyalty (0 (no) or 1 (yes))
Delgado-Ballester et al. (2014) [77]	To develop and test a conceptual model of the moderating effect of customers’ value consciousness on the relationship of store image with four dimensions of the perceived risk associated with the purchase of a PL over a manufacture brand, and the direct effect of those variables on the perceived unfairness of manufacture brand prices.	For each factor, a 10-point scale: SI: 7 items; FR: 3 items; FiR: 3 items; SR: 4 items; PR: 3 items; PU: 3 items; VC: 5 items
Bauer et al. (2013) [29]	To analyze if an organic labeled product generates positive consumer brand perceptions and, thus, influences consumers’ food buying intentions. To investigate how various types of brands’ benefit differently from organic labeling in the retail market.	Study 1: 12 German consumers using the laddering technique Study 2: 7-point Likert scale for 12 items: PH: 4 items; PHe: 4 items; EF: 4 items FS: 4 items Study 3: 7-point Likert scale for 2 items: PI: 1 item; WP: 1 item
Fall Diallo et al. (2013) [78]	To investigate how consumer and image factors, as well as store familiarity, influence PL purchase behavior.	7-point Likert scale for 24 items: SIP: 4 items; SB PI: 4 items; VC: 4 items; A: 4 items; PIn: 4 items; PL choice: 4 items
Herstein et al. (2012) [79]	To investigate the association between 3 personality traits (individualism, materialism, and the “need for cognition”) and 2 characteristics of shoppers who buy PLs, and the importance they attach to the “brand dimensions”.	5-point Likert scale: Study 1: 10 items (5 food and 5 non-food products) Study 2: 2 items Study 3: 33 items: VI: 4 items; HI: 4 items; M: 7 items; NC: 18 items
Wyma et al. (2012) [80]	To explore and describe consumers’ preferences for different PLs and national brands in a South African context. To determine and describe a possible relationship between consumers’ psychographic and demographic characteristics and their preferences for PLs/national brands.	25 items, choose the brand which fits one’s preference 5-point Likert scale 8 items + living standard measure
Tifferet and Herstein (2010) [81]	To analyze whether individualism affects consumers’ preference for PLs vs. national brands; assess the effect of individualism on the perceived importance of brand image dimensions (country of origin, packaging design, and manufacturer reputation); and assess the degree of cross-cultural differences in individualism.	5-point Likert scale: Study 1: 10 items Study 2: 30 items Study 3: 8 items
Glynn and Chen (2009) [82]	To examine the differences in the level category of risk perception and brand loyalty effects on consumer proneness towards buying PLs.	5-point Likert scale for 16 items: PM: 2 items; QV: 3 items; S vs. E: 2 items PC: 3 items; PQP: 3 items; BL: 3 items PL purchase: buy NBs (1) or PLs (5)
Anchor and Kourilová (2009) [83]	To show how relatively little is known about the consumer perceptions of PLs in the newly emerging markets of Central and Eastern Europe. To investigate various aspects of consumer perceptions of Tesco PLs in the Czech Republic.	3 items: 7-point semantic differential (SD) scale 2 brands x 4 items: 7-point SD scale
Kara et al. (2009) [84]	To examine consumers’ behavior with regard to PL purchasing by using a conceptual model, which incorporates factors such as brand, price and risk perceptions, involvement, experience, and familiarity, as well as psychographic and demographic factors.	27 items (5-point Likert scale)
Albayrak and Aslan (2009) [85]	To identify the attitudes toward PL products and demographic features of PL consumers and of manufacturer brand consumers. To determine whether any differences exist between the two consumer groups.	5-point Likert scale: 4 × 16 items
Cheng et al. (2007) [86]	To investigate the differences in the consumer perceptions of product quality, price, leadership, and personality brand among national brands, international and local PLs.	2 products x 3 types of brand x 4 items for 1 product 7-point Likert scale: PQ: 3 items BL: 3 items PP: 1 item BP: 3 items
Mieres et al. (2006) [87]	To analyze the effects that a set of variables related to purchasing behavior have on the difference in perceived risk between PLs and national brands.	Each item for kitchen rolls and shampoo: A: 7-point Likert scale: PQ: 4 items; REA: 7 items; SSC: 5 items FSB: 4 items; EPC: 4 items B: 7-point Likert scale: FR: 4 items; FiR: 3 items; SR: 4 items PR: 4 items; PsR: 4 items; TR: 4 items
Akbay and Jones (2005) [88]	To determine whether purchase patterns are differ for two income groups, and whether these differences are consistent with economic theory. To analyze the relationship between income and shopping behavior.	A: 1. 9 items: % scales 2. 9 items: cents per ounce B: 1. 9 items: % scales 2. 9 items: cents per ounce C: 1. 18 items: the LA/AIDS model 2. 18 items: the LA/AIDS model D: 1. 8 items: the LA/AIDS model 2. 18 items: the LA/AIDS model 3. 18 items: the LA/AIDS model
Kurtulus et al. (2005) [89]	To construct a model to determine the effect of the psychographics of consumers on their tendency to purchase PLs. To analyze the role of consumer attitudes and behaviors in consumer preferences for PLs.	5-point Likert scale: PC: 4 items; FC: 4 items QC: 4 items; SL: 4 items SM: 3 items; TL: 3 items BL: 3 items; T: 3 items
Semeijn et al. (2004) [90]	To investigate how store image and the perceived risk associated with product attributes affect the consumer evaluation of PLs. To determine the structural relationships between store image, the perceived risk associated with product attributes, and consumer attitude towards PLs.	Study 1: 11 items on a 7-point Likert scale Study 2: 7-point scale Study 3: 3 stores x 4 products 12 items: 7-point Likert scale
Veloutsou et al. (2004) [91]	To compare the importance of choice criteria when purchasing PLs and national brands, and the perceived characteristics of the products under PLs and manufacturer brands in two regions at different stages of PL development. To rate the change in the behavior towards PLs and supermarkets and product attributes (perceived quality, value for money, appealing packaging, perceived taste, and the importance of these values for PLs and national brands).	Study 1: 4 items on a 5-point Likert scale Study 2: average of the 5 categories of products; 5-point semantic differential scales (SEM) A: 4 items B: 5 items C: 5 items Study 3: 5 items on a 5-point SEM
Miquel et al. (2002) [92]	To model the decision process involved in a purchase when choosing PLs over national brands, and investigate why the same consumer may choose a store brand in one product category and not in another.	(1) 2 items: 5-point Likert scale (2) 2 items: 5-point Likert scale (3) 2 items: do not buy SB (0)/buy SB (1)
Vaidyanathan and Aggarwal (2000) [93]	To examine how a national brand’s extension to a PL product (through ingredient branding) affects the evaluation of national brands and PLs.	PA: 10 items on a 7-point SEM scale QP: 5 items on a 7-point quality scale VP: 6 items on a 7-point scale VC: 7 items on a 7-point value scale
